# A Combination of Real-Time PCR and High-Resolution Melting Analysis to Detect and Identify CpGV Genotypes Involved in Type I Resistance

**DOI:** 10.3390/v11080723

**Published:** 2019-08-06

**Authors:** Aurélie Hinsberger, Stéphane Theulier Saint Germain, Patrice Guerrero, Christine Blachère-López, Miguel López-Ferber, Sandrine Bayle

**Affiliations:** 1 LGEI, Ecole des Mines d’Alès, Institut Mines-Télécom et Université de Montpellier Sud de France, 6 Avenue de Clavières, 30100 Alès, France; 2 Ecole de l’ADN, 13 Boulevard Amiral Courbet, 30000 Nîmes, France; 3 INRA, 6 Avenue de Clavières, 30319 Alès, France

**Keywords:** *Cydia pomonella granulovirus*, codling moth, biological control, resistance, high resolution melting (HRM), *pe38* gene

## Abstract

*Cydia pomonella* granulovirus, in particular CpGV-M isolate, is used as a biological control against the codling moth (CM), *Cydia pomonella*. As a result of intensive control over the years, codling moth populations have developed resistance against this isolate. This resistance is now called type I resistance. Isolates, among them, CpGV-R5, have been found that are able to overcome type I resistance. Both CpGV-M and CpGV-R5 are used in orchards to control the codling moth. High resolution melting (HRM) has been adapted to differentiate between CpGV-M and CpGV-R5 isolates. Specific PCR primers have been designed for the CpGV *p38* gene, encompassing the variable region responsible for the ability to overcome resistance. Because each amplicon has a specific melting point, it is possible to identify the CpGV-M and CpGV-R5 genotypes and to quantify their relative proportion. This method has been validated using mixtures of occlusion bodies of each isolate at various proportions. Then, the HRM has been used to estimate the proportion of each genotype in infected larvae or in occlusion bodies (OBs) extracted from dead larvae. This method allows a rapid detection of genotype replication and enables the assessment of either success or failure of the infection in field conditions.

## 1. Introduction

In 2017, apple production represented around 5 × 10^6^ ha worldwide and pear production represented 1.39 × 10^6^ ha [1]. The codling moth (CM), *Cydia pomonella*, is a major pest in these orchards [2] and to control CM, on average, 35 treatments per year are required during the life span of the crop [3,4]. Intense chemical control leads to deleterious environmental consequences (i.e., azinphos-methyl, Guthion^®^ [5,6]), health risks (i.e., Chlorpyrifos, Lorsban^®^), but also CM resistance (dichlorodiphenyltrichloroethane (DDT) [7], anthranilic diamide insecticide chlorantranilipole [8], emamectin, tebufenozide [9] or organophosphorus [10]).

To reduce the chemical impact, biological alternatives have been studied, among them, isolates of a baculovirus, the *Cydia pomonella granulovirus* (CpGV). Baculoviruses have the following two phenotypes during the infection cycle which are different in structure and function: a budded virus (BV) which is a burgeoning form of the virus involved in systemic infection (cell-to-cell infection) and a virus occluded in a proteinaceous matrix, the occlusion body (OB), which allows survival in the environment and is responsible for between-host transmission. OBs dissolve in the insect larvae midgut releasing the virus particles that they contain, i.e., the, so-called, occlusion derived virus (ODV) that infects the midgut brush cells, starting the colonization of larval tissues [5].

The first CpGV isolate was discovered in Mexico and described by Tanada in 1964 [11]. It is called CpGV-M. Its complete genome is 123.5 kbps in length [12]. Soon after its discovery, other isolates were found around the world [13]. A classification of the CpGV genotypes into four (A to D) types was proposed [14] and CpGV-M belongs to the A type. A fifth type (E) was proposed later [15].

Bioinsecticides based on the CpGV-M isolate were registered by the 1990s in Europe for biological control of CM [5]. CpGV derived pesticides are registered in 34 countries worldwide [16]. In Europe, the orchard area using CpGV for the control of CM is estimated to be over 100,000 ha [17].

After years of use, failures to control orchards treated with CpGV-M based products were detected in Germany [18] and France [19], then in other countries (Austria, Czech Republic, Italy, Netherlands, and Switzerland [20]). Today, this resistance is called type I resistance [21].

Following the detection of the type I resistance, research was conducted to find virus isolates able to bypass it. Various isolates were found including CpGV-E2 [13], CpGV-I07 [22], CpGV-I12 [23], CpGV-S [16], CpGV-R1, and CpGV-R5 [24].

In type I resistant insects, CpGV-M replication is blocked at an early stage in all cells [25], while the other isolates replicate, although not all with the same efficiency [26]. It has been demonstrated that a modification of the virus ORF24 (*pe38* gene) is associated with the ability of virus isolates to replicate in insects carrying type I resistance [16]. Accordingly, it should be possible to predict if a given isolate is able to replicate in insects carrying type I resistance by analyzing its *pe38* gene. Currently, the following two reliable tools are available to characterize isolates: an agarose gel electrophoresis after polymerase chain reaction (PCR) amplification of the *pe38* gene [27] and sequencing of the gene [16].

Differences in melting points of a DNA fragment reflect differences in the DNA sequences, either in length or in base composition and when used at the whole genome level [28] this approach can be used to identify species. It has also been used to differentiate between variants on a given gene or gene fragment [29].

High resolution melting (HRM) allows a quick characterization of genetic variants based on the differences of the melting point of small DNA amplicons produced by PCR [30]. Each PCR product has a specific melting temperature and a unique melting curve [31]. HRM analysis enables the detection and sorting of amplicon sequences by differences in size [31,32,33,34] or in sequence, up to the detection of a single nucleotide polymorphism (SNPs). Moreover, HRM allows quantification of the relative proportions of each variant in mixed samples by using a standard range from DNA mixes.

Mixed CpGV infections can occur in a single larva. Analysis of the OBs extracted from a single larva collected in the field revealed the presence of two different genotypes [22], although the classical methods of analysis do not yet study the frequency of such events.

Detecting the frequency of the occurrence of such mixed infections in individual larva, and the relative frequency of the coinfecting genotypes is important in the design of more efficient biological control strategies. In the Nicaraguan isolate of Spodoptera frugiperda nucleopolyhedrovirus (SfMNPV-Nic), it has been observed that multiple infections increase the efficacy of the virus population [35]. A recent study reported observing a higher than expected efficacy of CM control by mixtures of CpGV-R5 and CpGV-M [27].

In this study, the HRM method has been adapted to identify two viral genotypes, group A (CpGV-M) and group E (CpGV-R5) (that are the major components of the Carpovirusine^®^ Evo2) and their proportion within infected insects. This approach could be easily applied to monitor the infection status of large samples of codling moth larvae in treated orchards.

## 2. Materials and Methods

### 2.1. Insects

CpNPP was our reference laboratory colony susceptible to CpGV-M. It originated from the Northern part of France, and it has been maintained in a laboratory for almost 30 years. It is used for the industrial production of Carpovirusine^®^, and was provided by the Natural Plant Protection SA (Pau, France) [27].

### 2.2. Viruses

A single stock of CpGV-M [11] (our laboratory stock 2020-s1) was used in all experiments. The CpGV-R5 isolate has been described previously [36]. The 2016-r16 stock of this virus isolate was used in all experiments. CpGV-M and CpGV-R5 were adjusted to the same concentration from a primary quantification by qPCR.

The AcMNPV clone 1.2 used for negative control of PCR was provided by CNRS UPS3044 Baculovirus and Therapy. (GenBank accession numbers L22858.1) [37].

### 2.3. Amplification of the Different Viral Mixed Populations

Fifty µL of a viral suspension at a concentration of 800 OBs/µL were deposited on the surface of 24-well plates filled with approximately 1 g of medium (StoneflyHeliothis Diet, Ward’s Science, Rochester, NY, USA). Third-instar larvae (L3) (7 days old) were placed onto the surface. The plates were incubated at 25 °C (±1 °C) with a 16:8 h (light/dark) photo period and a relative humidity of 60% (±10%).

After 4 days, diseased larvae were extracted from the medium and kept at 25 °C for one more day in 1.5 mL microfuge tubes. Infected larvae were then crushed into distilled water. The mixture was filtered through nylon to remove insect debris. Then, the suspension was centrifuged 5 min at 8000 g, and the pellet was resuspended in distilled water (1 mL for 10 larvae).

The final virus stocks (concentration around 10^11^ OB/mL) were stored at −20 °C [24,38].

### 2.4. Infection Categories

#### 2.4.1. Standard Range for Quantification in HRM Analysis

First, the OB concentrations of the viral stocks were pre-estimated as previously described by [4]. CpGV-M and CpGV-R were purified by QIAamp^®^ (Section 2.5). Then, the virus DNA content of 2020-s1 stock (CpGV-M) and 2016-r16 stock (CpGV-R5) was determined by qPCR to precisely adjust the relative proportions of the two genotypes.

Each mix was identified by the relative proportions of each genotype, “M*i*” and “R*j*”, *i* being the frequency of CpGV-M and *j* being the frequency of CpGV-R5 on the inoculum. Eleven virus mixes were prepared with different proportions of pure isolate DNA: CpGV-M (M100-R0) and CpGV-R5 (M0-R100), 99% CpGV-M + 1% CpGV-R5 (M99-R1), 95% CpGV-M + 5% CpGV-R5 (M95-R5), 90% CpGV-M + 10% CpGV-R5 (M90-R10), 75% CpGV-M + 25% CpGV-R5 (M75-R25), 50% CpGV-M + 50% CpGV-R5 (M50-R50), 25% CpGV-M + 75% CpGV-R5 (M25-R75), 10% CpGV-M + 90% CpGV-R5 (M10-R90), 5% CpGV-M + 95% CpGV-R5 (M5-R95), and 1% CpGV-M + 99% CpGV-R5 (M1-R99). DNA mixes were analyzed by qPCR and HRM in triplicate (Section 2.7).

#### 2.4.2. Viral Infections of CpNPP with Mixes and Analysis of BV Production

CpNPP larvae were individually infected with M0-R100, M25-R75, M50-R50, M75-R25, and M100-R0 OB mixes. Seventy-two hours post inoculation the larvae were washed in 0.1% SDS and rinsed twice in water. The hemolymph of each larva was recovered individually with slender Pasteur pipette and used as a template for the analysis, represented by H(M*i*-R*j*). Two randomly chosen larvae were analyzed for each of the five OB mixes. Melting curves were compared to the standard range (Section 2.4.1).

#### 2.4.3. Viral Infections of CpNPP with Mixes and Analysis of OBs Production

Twenty CpNPP larvae were individually infected with six OB mixes (M100-R0, M99-R1, M90-R10, M50-R50, M10-R90, and M0-R100). Four days after infection, the diseased larvae were collected collectively and crushed and filtered as above (Section 2.3). The OBs produced on these infections were named P(M*i*-R*j*). OBs were purified by QIAamp^®^ (Section 2.5) and analyzed by qPCR and HRM (Section 2.7). Melting curves were compared to the standard range (Section 2.4.1).

### 2.5. DNA Purification

DNA was purified using a QIAamp^®^ DNA Mini Kit (Qiagen, Hilden, Germany) according to the conditions recommended.

### 2.6. Primer Design

The primers were designed using Clone manager V9 (SCI-ED software, Denver, CO, USA). The primers were synthesized by Eurofins MWG (Ebersberg, Germany). The forward primer CpGV-18734F (5′-GCCACCATTAGTGAATCATC-3′) and the reverse primer CpGV-18855R (5′-TAAGTCAGGACACCCAAACC-3′) were designed to target the variable region of ORF 24, gene *pe38*, and to encompass the 24 bps deletions. The resulting amplicons for CpGV-M and CpGV-R5 were 121 and 97 bps in length.

### 2.7. HRM Assay

qPCR was performed in triplicate with 200 nM of each primer (best concentration tested, data not shown), 4 µL of 5× HOT FIREPol^®^ EvaGreen^®^ HRM Mix (no ROX) (Solis Biodyne, Tartu, Estonia), 5 µL of viral DNA, and water up to 20 µL final.

The PCR amplification and HRM analysis were performed using a CFX96 Touch™ Real-Time PCR Detection System (Bio-Rad Laboratories, Hercules CA, USA) in Hard-Shell^®^ 96-Well PCR Plates, white and clear. The program of the thermocycler was 15 min GoTaq activation at 95 °C, 15 s denaturing at 95 °C, 40 s annealing at 55 °C, and 30 s extension at 72 °C for 40 cycles. An additional step was added, i.e., 5 s at 95 °C, then 10 s at 50 °C. After the PCR amplification, the high-resolution melting analysis was performed by increasing the temperature from 70 to 90 °C in steps of 0.2 °C maintained for 10 s each [33].

The data was analyzed using the Biorad CFX maestro™ for qPCR interpretation and Precision Melt Analysis™ Software for HRM parameters determination (Bio-Rad Laboratories). OB and BV production from mixes (Section 3.2 and Section 3.3) infections were compared to the standard range (Section 3.1).

### 2.8. Amplicon Sequencing

PCR amplicons were sequenced by Eurofins MWG (Ebersberg, Germany). The sequences obtained were aligned using Clone manager V9 (SCI-ED software).

## 3. Results

### 3.1. Identification and Quantification of Genotypes in a Mixture

**Figure 1:**
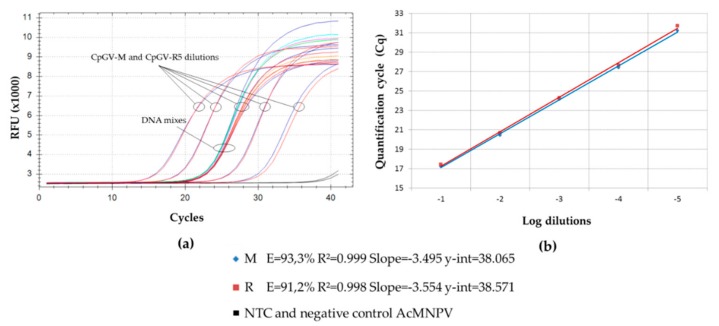
PCR amplification of CpGV *pe38* gene for a range of dilutions of CpGV-M and CpGV-R5, and DNA mixes. (**a**) Relative fluorescence unit (RFU) upon PCR cycles. DNA mixes are encompassed by that of the reference virus dilutions tested. Amplifications from CpGV-M and CpGV-R5 are almost superposed for the whole range of dilutions tested. (**b**) Quantification cycle (Cq) depending on serial dilution for CpGV-M and CpGV-R5. Efficacy (E), correlation coefficient (R^2^), and y-intercept (y-int) are shown for pure CpGV-M and pure CpGV-R5.

The PCR amplification of amplicons within the *pe38* gene was performed in triplicate on pure genotype virus using the primers CpGV-18734F and CpGV-18855R. Both M100-R0 and M0-R100 were used as references. The efficacies of PCR were around 91% and 93%, for CpGV-M and CpGV-R5, respectively, and the value of the y-intercept was 38 (Figure 1). These efficacies were close enough to allow quantification of each genotype in a mixture. All no template controls (NTCs) got a quantification cycle (Cq) value higher than the y-intercept (NTC greater than 5 Cq). The nonspecific amplification in NTCs was due to dimer formation at the end of the cycle. Accordingly, it could be excluded from the analysis. Two negative controls were added, i.e., an uninfected larva and a different baculovirus, AcMNPV1.2. No amplification was obtained with the controls that behaved as NTC.

The *pe38* amplicons of CpGV-M (M100-R0) and CpGV-R5 (M0-R100) were checked by sequencing. The sequences obtained correspond to the published ones [36]. The same deletion of 24 bps described by Gebhardt et al. [16] was observed between CpGV-M and CpGV-R5.

**Figure 2:**
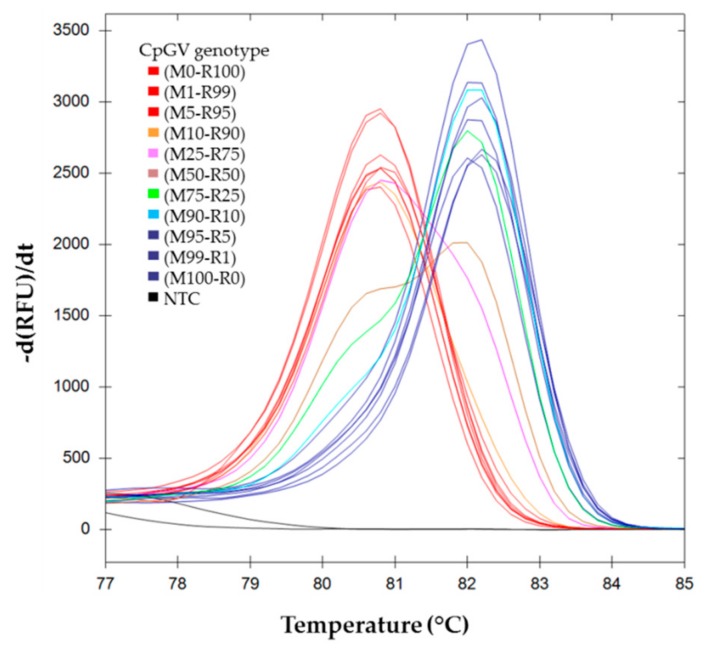
Melting curves analysis of *pe38* amplicons in DNA mixture. First derivative of RFU (relative fluorescence unit) depending on temperature are represented for each inoculum mix M*i*-R*j*, where M*i* indicates the relative proportion of CpGV-M and R*j* indicates the relative proportion of CpGV-R5. (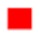) (M0-R100), (M99-R1), (M95-R5); (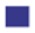) (M100-R0), (M1-R99) and (M5-R95); (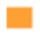) (M90-R10); (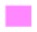) (M75-R25); (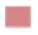) (M50-R50); (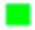) (M25-R75); (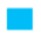) (M10-R90).

The results obtained for the melting curves for M100-R0, M99-R1, M95-R5, M90-R10, M75-R25, M50-R50, M10-R90, M25-R75, M5-R95, M1-R99, and M0-R100 are shown in Figure 2. The CpGV-R5 pure genotype (M0-R100) (mean tm = 80.80, SD = 0.00), and the CpGV-M pure genotype (M100-R0) (mean tm = 82.13, SD = 0.12) show single peaks. Mixed populations show two peaks, which positions correspond to each genotype, and the intensity ratios are proportional to the genotype proportions.

**Figure 3:**
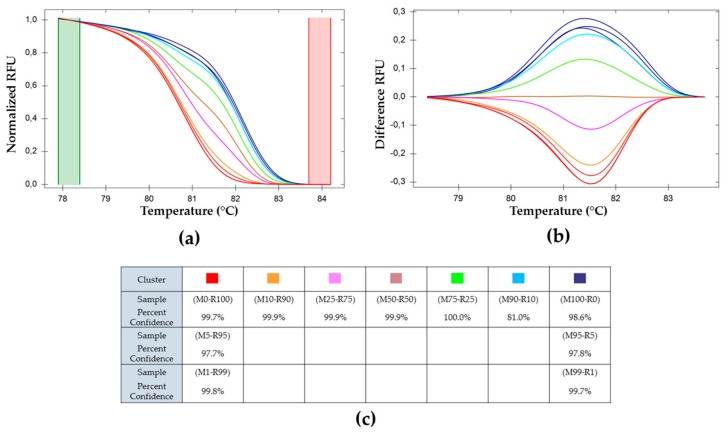
Melting curve analysis of *pe38* amplicons in DNA mixtures. (**a**) Normalized relative fluorescence unit (RFU) variation with temperature, (**b**) plot of deviations of RFU related to M50-R50 used as a reference with varying temperatures and (**c**) percent of confidence for each cluster. (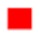) (M0-R100), (M99-R1), and (M95-R5); (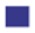) (M100-R0), (M1-R99) and (M5-R95); (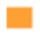) (M90-R10); (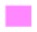) (M75-R25); (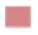) (M50-R50); (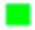) (M25-R75); (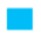) (M10-R90).

The genotypes have been grouped automatically into HRM profile clusters by the software Precision Melt Analysis^TM^. Each color represents one cluster. Fluorescence intensity data, shown in Figure 2, are normalized and reduced (values ranging between zero and one) using values both before and after the melting phase. The software clusters the samples by calculating their probability to belong to the same cluster based on their standard deviation and their mean melting curve [39]. The cutoff level for clustering was set to α = 0.05. The 11 samples are aggregated in seven clusters (Figure 3). Figure 3a represents the normalized data, characteristic of a given mix of genotypes. To improve the representation of the results, data was plotted as the difference to the M50-R50 cluster (Figure 3b) [39]. In such a representation, the extreme differences are those of the pure genotypes M100-R0 and M0-R100. When the frequency of one of the genotypes is higher than 90%, the software clusters it as the pure genotype (same color). The percent of confidence represents the relative probability for a sample to belong to a cluster, as shown in Figure 3c. The clustering confidence is lower in the samples that are wrongly associated than in those correctly clustered, indicating that the within-group variability is higher in the former.

On the positive side of the vertical axis, mixes with predominantly CpGV-M genotype are plotted; on the negative side, those with majority CpGV-R5, with the M100-R0 and M0-R100 at the extremities. These mixes are used as standard range in the estimation of the relative abundance of genotypes in virus infections. The range of the difference curve is proportional to the frequency of genotypes in that mix.

### 3.2. Analysis of BVs Extracted from Hemolymph

Larvae were fed with different OB mix inoculums. The hemolymph of these larvae were individually collected and used as a source for the BV. The PCR amplifications were performed as above. Two randomly chosen larvae (a and b) were analyzed for each condition [H(M*i*-R*j*)].

**Figure 4:**
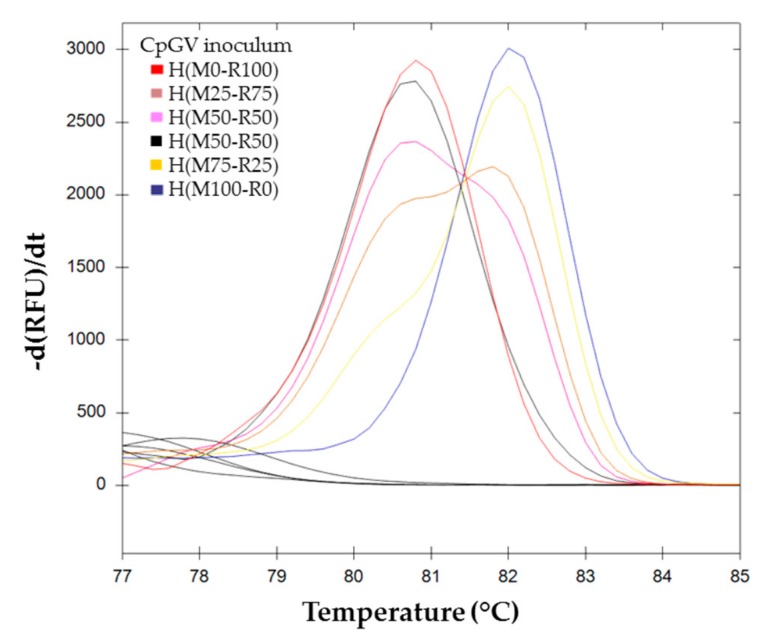
Melting curve analysis of *pe38* amplifications using BVs obtained on larvae in conditions of mixed infection. (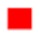) H(M0-R100), (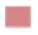) H(M25-R75), (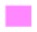) H(M50-R50)a, (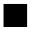) H(M50-R50)b, (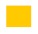) H(M75-R25), and (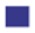) H(M100-R0).

The melting curves for BVs resulting from pure and mixed infection H(M100-R0), H(M75-R25), H(M50-R50), H(M10-R90), and H(M0-R100) are shown in Figure 4.

**Figure 5:**
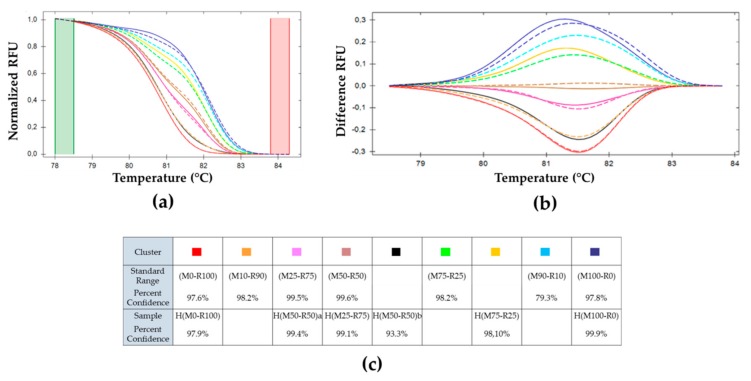
Melting curves analysis of *pe38* amplifications using BVs obtained on larvae in conditions of mixed infection and compared to the standard range. (**a**) Normalized RFU. (**b**) First derivative of fluorescence vs. temperature profile, cluster (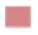) (M50-R50) of the standard range is used as reference. Cluster of the standard range is represented with dotted lines: (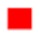) (M0-R100); (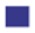) (M100-R0); (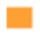) (M10-R90); (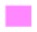) (M25-R75); (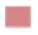) (M50-R50); (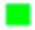) (M75-R25); (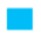) (M90-R10). BVs resulting from each inoculum: (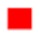) H(M0-R100); (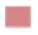) H(M25-R75); (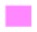) H(M50-R50)a; (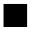) H(M50-R50)b; (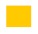) H(M75-R25) and (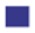) H(M100–R0). (**c**) Percent of confidence for each sample belonging to a cluster.

The melting curves of these samples were compared to the standard range obtained previously (Section 3.1). The sample values are plotted in solid lines while the standard range curves are represented with dotted lines (Figure 5). H(M100–R0) and H(M0-R100) were used as positive controls. They were associated in the same cluster as M100-R0 and M0-R100 of the standard range.

The two samples H(M50-R50)a and H(M50-R50)b clustered independently from each other; while the two H(M75-R25) samples fell into the same cluster. Of the two H(M25-R75) samples, only one yielded amplification, but did not cluster with the parental M25-R75.

### 3.3. Analysis of OBs Productions

**Figure 6:**
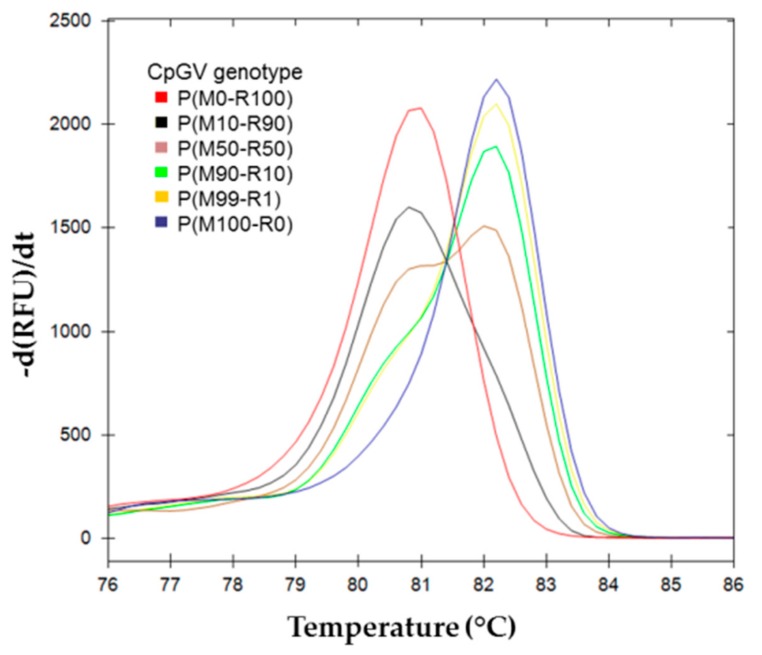
Melting curves analysis of *pe38* amplicons from CpGV OBs extracted from infected larvae. First derivative of RFU (relative fluorescence unit) upon temperature profile are represented for each OB production analyzed. (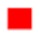) P(M0-R100); (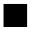) P(M10-R90); (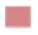) P(M50-R50); (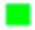) P(M90-R10); (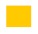) P(M99-R1); (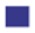) P(M100-R0).

Larvae were allowed to feed on various inoculums, including the pure genotypes, and the resulting OBs [P(M*i*-R*j*)] were analyzed by HRM. The results obtained for the melting curves of these OBs are shown in Figure 6.

**Figure 7:**
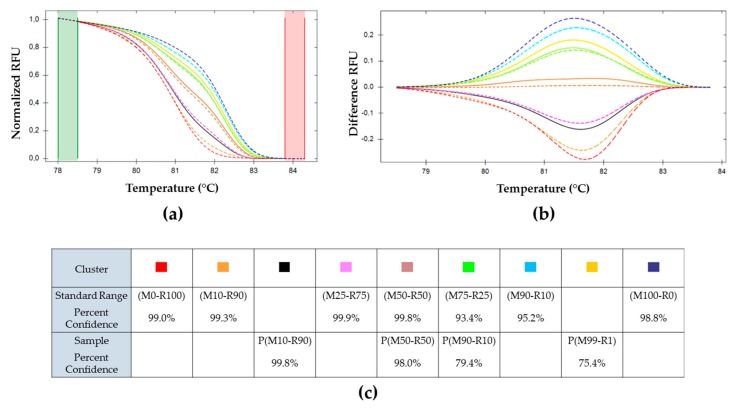
Melting curves analysis of *pe38* amplifications of OBs production after multiple infection as compared with the standard range. (**a**) Normalized relative fluorescence unit (RFU). (**b**) Difference RFU upon temperature, (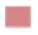) (M50-R50) used as reference. Cluster of the standard range is represented in dotted lines: (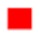) (M0-R100), (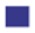) (M100-R0), (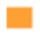) (M10-R90), (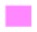) (M25-R75), (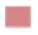) (M50-R50), (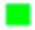) (M75-R25), and (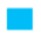) (M90-R10). OBs resulting from each inoculum: (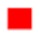) P(M0-R100), (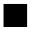) P(M10-R90), (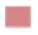) P(M50-R50), (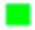) P(M90-R10), (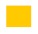) P(M99-R1), and (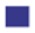) P(M100-R0). (**c**) Percent of confidence for each sample to belong to a cluster.

The HRM determination and data analysis were done similarly to the previous experiments. The sample values are plotted in solid lines while the standard range curves are represented with dotted lines (Figure 7). P(M100-R0), P(M50-R50), and P(M0-R100) infections were associated by HRM software in the same clusters as their corresponding parental inoculum in the standard range (M100-R0), (M50-R50), and (M0-R100), respectively. The other OBs productions did not cluster with their respective parental references. P(M10-R90) was recognized as a new cluster, it was between (M10-R90) and (M25-R75) (closer to (M25-R75) of the standard range). P(M90-R10) was associated to (M75-R25).

## 4. Discussion

Gebhardt et al. (2014) demonstrated that the difference of the *pe38* virus gene is necessary and sufficient to explain the difference of behavior of the two genotypes in insects carrying type I resistance. The main difference between *pe38* of CpGV-M and other CpGVs is a short 24 bps insertion in the former [16].

In previous work, we took advantage of this difference of size to differentiate genotypes by PCR, using a pair of PCR primers, CpGV-18705F and CpGV-19003R [27]. They amplify a 295 and 315 bps fragment on CpGV-M and CpGV-R5, respectively. These fragments could then be separated by agarose gel electrophoresis. However, because this approach is labor intensive it does not allow processing a large number of samples. Moreover, it prohibits the determination of each genotype proportion in a virus mix.

In a recent paper by Krejmer-Rabalska et al. (2019), several granuloviruses were discriminated by qPCR based on the amplification of three conserved genes, *lef-8*, *lef-9*, and *gran* [29]. Their approach, however, was not meant to evaluate the relative proportions of different genotypes within the same virus species.

The development of the PCR, and the increasing precision of the analytical systems today, allow detection of point mutations in short DNA fragments after PCR amplification. The high resolution melting point analysis of these PCR fragments is thus a convenient way for detecting small differences between DNA sequences [31], providing that appropriate specific primers can be developed. Accordingly, an easy differentiation between the two genotypes, CpGV-M and CpGV-R5, could be obtained. A pair of primers that specifically amplifies the required region was developed. CpGV-18734F and CpGV-18855R amplify fragments of 121 for CpGV-M and 97 bps for CpGV-R5. The mean melting temperatures obtained for each genotype differed by 1.3 °C. As the system has a 0.15 °C resolution, it was possible to resolve the peaks in mixed genotype samples (Figure 2). The final weight of the peak depends on the frequency of the template, and on the efficacy of the amplification for each template. Our experimental conditions were set up in such a way that both templates amplify with similar efficacy (93.3% and 91.2% for CpGV-M and CpGV-R5, respectively) (Figure 1). As a consequence, the importance of each peak correlates with the frequency of the template, allowing estimation of its frequency.

The ability of HRM to use these fusion curves to cluster samples as a function of the proportion of each genotype was checked on a standard range set obtained from the pure CpGV-M and CpGV-R5 genotypes, and from the DNA mixes of the following genotypes in various proportions: M100-R0, M90-R10, M50-R50, M10-R90, M1-R99, and M0-R100. It was possible to discriminate between all the mixes except M1-R99, M5-R95, and M0-R100 that are grouped in the same cluster (Figure 3). Using this approach, it was, thus, possible to quantify the relative proportions of these two genotypes except when the frequency of one of the genotypes was less than 10%.

To facilitate the analysis, the HRM output was subjected to normalization and reduction, allowing comparisons between experiments. In addition, by plotting the difference of the whole fusion curve against the M50-R50, the clusters were easily identified (Figure 3b, Figure 5b and Figure 7b). The composition of an unknown sample fell between the reference graph, making the estimation of its genotype frequencies possible.

As the issue of an infection of different larvae feeding on the same mixture of genotypes might not be identical to the parent genotypes, to complete the analysis, the following two tests were carried out: (i) using the hemolymph of infected larva directly and (ii) using the OBs progeny of such infections.

When analyzing the infection of hemolymph of an individual larva feeding on the same mixed genotype sample, the results were not always identical, as shown in Figure 5 for two larvae fed on M50-R50. This possibly reflects the addition of variability on feeding (sampling variability) and on the development of the infection (susceptibility variability). These deviations might also reflect a selection for one genotype, although in our previous work [27] we did not detect selective advantages on the traits that were measured (OB production, infectivity). As a consequence, when analyzing samples of OBs progeny of mixed infections (Figure 7), deviations from the inoculation ratios were also observed. Further studies would be required to follow these frequency variations over passage at the individual level. They could shed light on the eventual selection of one or another genotype.

The CM populations carrying the type I resistance are found in seven countries throughout Europe. Apple growers rely on the analysis of the resistance made by analysis of the offspring of diapausing insects collected in the previous year. In addition to being time consuming, this approach cannot be applied to each orchard and, thus, it is impossible to evaluate the success or the failure of a treatment in each field at each generation.

Various virus-based biocontrol products are available in the market. Some of them are based exclusively on the CpGV-M isolate, while others, that are effective against insects carrying the type I resistance, as well as susceptible insects [26], appeared to be composed by mixtures of virus genotypes belonging to different groups. Among them, Carpovirusine^®^ Evo2 contains 25% A type (CpGV-M) and 60% E type (CpGV-R5) [40].

As replication of CpGV-M alone cannot occur in larvae carrying type I resistance, the proportion of PCR negative larvae will be a good estimate of the prevalence of the type I resistance when an orchard is treated with a product containing only CpGV-M. Using such a test on the first generation will allow changing the product at the second generation, if required. Conversely, if pure CpGV-M infections are detected after treatment with products containing both genotypes, not all CM larvae in the orchard are resistant, i.e., the resistance gene has not been fixed. Hypothetically, if no CpGV-R5 infected larvae are found when treating with a mixture of OB genotypes, it would reveal a new type of resistance specifically blocking type E genotypes, however, such a resistance has not yet being found.

This tool provides an easier way to analyze the infection status of larvae feeding on orchards that have been treated with CpGV-based products, therefore, the success or failure of the treatment at an early stage can be anticipated, offering the possibility for the grower to use alternative approaches.
